# Asiatic acid attenuates hypertrophic and fibrotic differentiation of articular chondrocytes via AMPK/PI3K/AKT signaling pathway

**DOI:** 10.1186/s13075-020-02193-0

**Published:** 2020-05-12

**Authors:** Na Liu, Dejie Fu, Junjun Yang, Pingju Liu, Xiongbo Song, Xin Wang, Rui Li, Zhenlan Fu, Jiajia Chen, Xiaoyuan Gong, Cheng Chen, Liu Yang

**Affiliations:** 1 Key Laboratory of Freshwater Fish Reproduction and Development, Ministry of Education, Laboratory of Molecular Developmental Biology, School of Life Sciences, Southwest University, Chongqing, China; 2 Center for Joint Surgery, Southwest Hospital, Third Military Medical University (Army Medical University), Chongqing, China; 3 Zunyi Traditional Chinese Medicine Hospital, Zunyi, China; 4 Biomedical Analysis Center, Third Military Medical University (Army Medical University), Chongqing, China; 5 College of Medical Informatics, Chongqing Medical University, Chongqing, China

**Keywords:** Asiatic acid, Chondrocytes, Osteoarthritis, Hypertrophy, Fibrosis, AMPK, PI3K, AKT

## Abstract

**Background:**

Osteoarthritis (OA), the most common joint disorder, is characterized by a progressive degradation of articular cartilage. Increasing evidence suggests that OA is closely associated with cartilage pathologies including chondrocyte hypertrophy and fibrosis.

**Methods:**

In this study, we showed that asiatic acid (AA) treatment reduced chondrocyte hypertrophy and fibrosis. First, the cytotoxicity of AA (0, 5, 10, and 20 μM) to chondrocytes was evaluated, and 5 μM was selected for subsequent experiments. Then, we detected the gene and protein level of chondrocyte hypertrophic markers including type X collagen (COL-X), matrix metalloproteinase-13 (MMP-13), alkaline phosphatase (ALP), and runt-related transcription factor 2 (Runx2); chondrocyte fibrosis markers including type I collagen (COL-Ι) and alpha-smooth muscle actin (α-SMA); and chondrogenic markers including SRY-related HMG box 9 (SOX9), type II collagen (COL-II), and aggrecan (ACAN). Further, we tested the mechanism of AA on inhibiting chondrocyte hypertrophy and fibrosis. Finally, we verified the results in an anterior cruciate ligament transection (ACLT) rat OA model.

**Results:**

We found that AA treatment inhibited the hypertrophic and fibrotic phenotype of chondrocytes, without affecting the chondrogenic phenotype. Moreover, we found that AA treatment activated AMP-activated protein kinase (AMPK) and inhibited phosphoinositide-3 kinase/protein kinase B (PI3K/AKT) signaling pathway in vitro. The results in an ACLT rat OA model also indicated that AA significantly attenuated chondrocyte hypertrophy and fibrosis.

**Conclusion:**

AA treatment could reduce hypertrophic and fibrotic differentiation and maintain the chondrogenic phenotype of articular chondrocytes by targeting the AMPK/PI3K/AKT signaling pathway. Our study suggested that AA might be a prospective drug component that targets hypertrophic and fibrotic chondrocytes for OA treatment.

## Introduction

Chondrocyte hypertrophy plays an essential role in endochondral ossification during bone formation and growth [1]. However, ectopic hypertrophy of articular chondrocytes has been recognized as an osteoarthritis (OA)-promoting factor [2–4]. In early stages of OA, articular chondrocytes respond to the degenerative events by decreasing the production of type II collagen (COL-II) and increasing the synthesis of type X collagen (COL-X), which is an indication of chondrocyte hypertrophy [5]. Meanwhile, activity of matrix metalloproteinase-13 (MMP-13), a major collagenase expressed in hypertrophic chondrocytes, has been observed with OA onset [6]. In addition, a significantly increased expression of alkaline phosphatase (ALP) and runt-related transcription factor 2 (Runx2) is identified in osteoarthritic chondrocytes [7, 8]. Kamekura et al. investigated the functional involvement of Runx2 in chondrocyte hypertrophy and OA development and found that compared with wild-type mice, Runx2-deficient mice had no hypertrophic chondrocytes and showed a decrease in cartilage destruction, along with a reduction in COL-X and MMP-13 expression [9]. Therefore, chondrocyte hypertrophy is closely related to OA pathogenesis.

On the other hand, dedifferentiation of articular chondrocytes usually occurs during in vitro culture and expansion [10], which is characterized by the loss of COL-II and increase in type I collagen (COL-I) [11, 12]. The phenotypic instability and easy access to the fibroblast-like phenotype seriously affect the outcome of cartilage repair when using chondrocytes as seed cells. Taken together, how to inhibit chondrocyte hypertrophy and chondrocyte dedifferentiation is of great concern in both OA treatment and cartilage tissue engineering.

Asiatic acid (AA), a pentacyclic triterpene isolated from *Centella asiatica* [13], has been reported to exhibit a variety of pharmacological effects, including antioxidant, anti-inflammatory, and hepatoprotective activities [14–16]. Particularly, recent studies demonstrate that AA inhibits cardiac hypertrophy [17] and liver fibrosis [18]. However, whether AA could attenuate the hypertrophic differentiation or the fibrotic differentiation of articular chondrocytes has not been reported. We hypothesized that AA might attenuate chondrocyte hypertrophy or chondrocyte dedifferentiation. To verify this hypothesis, first we treated human osteoarthritic chondrocytes with AA and measured the changes of hypertrophic markers and fibrotic markers; then, we intra-articularly injected AA in a rat OA model and analyzed the joint histology after 4 weeks and 8 weeks.

## Materials and methods

### Chemicals

Asiatic acid (purity > 97.0%; molecular weight 488.70), purchased from Sigma–Aldrich (St. Louis, USA), was dissolved in dimethylsulfoxide (DMSO) as a 2-mM stock solution and stored at 4 °C. Further dilution was done in cell culture medium.

### Cell isolation and culture

Cartilage samples were obtained intraoperatively from patients (*n* = 3, age 55 ± 10) undergoing total knee arthroplasties with approval from the Ethics Committee of Southwest Hospital (Chongqing, China). Chondrocytes were isolated according to our previous protocol [19]. Briefly, cartilage pieces were digested in high-glucose DMEM (C11995500BT, Gibco, USA) supplemented with 0.2% type II collagenase (C6885, Sigma, USA) and 1% penicillin/streptomycin (P/S) overnight at 37 °C. The resulting cell suspension was filtered through a 40-μm cell strainer; collected cells were centrifuged (400*g* for 5 min) and resuspended in high-glucose DMEM supplemented with 10% fetal bovine serum (FBS; Hyclone, USA) and 1% P/S. Finally, cells were plated at a density of 1 × 10^5^ cells per well in 6-well plates and incubated in a humidified atmosphere of 5% CO_2_ at 37 °C. The medium was changed every 2–3 days. Only cells at passage 1 were used in our study to avoid phenotype loss.

### Live-dead cell staining and cell viability assay

The effects of AA on the viability of chondrocytes were evaluated using a Live/Dead staining kit (40747ES76, Yeasen, China). Briefly, after 24 h treatment of AA (0, 5, 10, and 20 μM), chondrocytes were incubated with 2 μM Calcein-AM and 4.5 μM PI for 15 min at room temperature (RT) in the dark. Labeled cells were visualized using a confocal microscope (IX71, Olympus, Japan). Live cells were stained green, whereas dead cells were stained red.

To further evaluate the cytotoxicity of AA, measurement of cell viability was performed using the Cell Counting Kit-8 (CCK-8; CK04, Do Jindo Laboratories, Japan). Chondrocytes were cultured in 96-well plates at a density of 5 × 10^3^ cells per well for 24 h. Then, cells were pretreated with AA at different concentrations (0, 5, 10, and 20 μM) for 24 h. After that, 10 μL CCK-8 solution was added to each well and incubated at 37 °C for 2 h. The optical density was read at a wavelength of 450 nm with a microplate reader (Thermo Fisher Scientific, USA).

### Alcian Blue staining

The cells were washed with PBS and fixed with 4% formaldehyde for 10 min at RT. Then, the cells were washed three times with PBS and stained with Alcian Blue (Cyagen, USA) for 30 min. The cells were washed again three times with PBS and imaged.

### Alkaline phosphatase staining

Cells were cultured in 24-well plates at a density of 1 × 10^4^ cells per well, followed by stimulation with AA. After 3 days of culturing, the cells were washed with PBS and stained using an ALP staining kit (C3206, Beyotime, China) according to the manufacturer’s protocol. The cells were washed again three times with PBS and imaged.

### Immunofluorescence staining

Cells were washed with PBS and fixed with 4% formaldehyde for 10 min at RT. Then, cells were washed three times with cold PBS and treated with Triton X-100 (P0096, Beyotime) for 10 min at RT. Cells were washed again three times with PBS and blocked 1 h with Blocking Buffer (P0260, Beyotime) at RT followed by incubation with the primary antibodies: COL-II (dilution of 1:200; ab34712, Abcam, USA), Aggrecan (dilution of 1:100; ab3778, Abcam), SOX9 (dilution of 1:250; ab185230, Abcam), MMP-13 (dilution of 1:200; ab39012, Abcam), COL-X (dilution of 1:50; ab58632, Abcam), Runx2 (dilution of 1:500; ab23981, Abcam), COL-Ι (dilution of 1:200; ab34710, Abcam), and α-SMA (dilution of 1:500; A5228, Sigma) overnight at 4 °C. Next, the cells were washed three times with PBST, incubated with secondary antibody for 1 h at RT. Cell nuclei were counterstained with DAPI for 5 min at 37 °C, and the images were obtained by confocal fluorescent microscope (LSM710, Carl Zeiss, Germany).

### Reverse transcription-polymerase chain reaction

RT-PCR total RNA was isolated from chondrocytes using RNA pure Total RNA Kit9 (RP5612, BioTeke, China) following the manufacturer’s protocol. RNA concentration was determined spectrophotometrically using a NanoDrop ND1000 spectrophotometer (Isogen Life Science B.V., Netherlands). The A260/A280 ratio was calculated to verify quality and purity. cDNA synthesis was performed using first strand cDNA synthesis kit (Roche, Switzerland) according to the manufacturer’s instructions. Real-time PCR was performed in 20 μL reactions on cDNA with SYBR Green PCR reagents (Roche) using CFX96 Touch TM Real-Time PCR Detection System (Bio-Rad, USA). The level of target mRNA was normalized to the level of GAPDH (B661104, Sangon Biotech, China) and compared with control. Data were analyzed using 2^−ΔΔCT^ method. Each gene analysis was performed in triplicate. Primer’s sequences of the targeted genes are listed in Table 1.

**Table 1:** Sequences of the primers used in this study

Name	Forward	Reverse
COL2a1	TGCTGCCCAGATGGCTGGAGGA	TGCCTTGAAATCCTTGAGGCCC
ACAN	TCCTGGTGTGGCTGCTGTCC	TCTGGCTCGGTGGTGAACTCTAG
SOX9	GACTTCCGCGACGTGGAC	GTTGGGCGGCAGGTACTG
MMP-13	TCCTGGCTGCCTTCCTCTTCTTG	AGTCATGGAGCTTGCTGCATTCTC
Runx2	AACAGCAGCAGCAGCAGCAG	GCACCGAGCACAGGAAGTTGG
COL10a1	GCCACCAGGCATTCCAGGATTC	GGAAGACCAGGCTCTCCAGAGTG
COL1a1	GCGAGAGCATGACCGATGGATTC	GCCTTCTTGAGGTTGCCAGTCTG
α-SMA	TCGTGCTGGACTCTGGAGATGG	CCGATGAAGGATGGCTGGAACAG
GAPDH	ACGGATTTGGTCGTATTGGG	CGCTCCTGGAAGATGGTGAT

### Western blot

The cells were lysed in RIPA (P0013B, Beyotime) with 1% PMSF (ST506, Beyotime) on ice for 5 min and removed with a scraper. Then, the lysate was centrifuged at 16,100*g* for 5 min, and the supernatant was collected. The samples were diluted with SDS-PAGE Sample Loading Buffer (Beyotime) and kept at 100 °C for 10 min. Proteins were separated in 8% to 12% sodium dodecyl sulfate polyacrylamide gel electrophoresis (according to the molecular weights) and transferred to a PVDF membrane (FFP28, Beyotime) at 200 mA for 1.5 h at 4 °C. The membrane was washed twice with Milli-Q water, stained with Ponceau S for protein visualization, and washed three times with 2% v/v TBST (tris-buffered saline with Tween-20). The blot was blocked with QuickBlock™ Blocking Buffer (P0228, Beyotime) for 2 h at RT and incubated separately with the following primary antibodies: COL-II (dilution of 1:5000), Aggrecan (dilution of 1:100), SOX9 (dilution of 1:5000), MMP-13 (dilution of 1:3000), COL-X (dilution of 1:250), Runx2 (dilution of 1:1000), COL-Ι (dilution of 1:5000), α-SMA (dilution of 1:1000), p-AMPK (dilution of 1:1000, AF3423, Affinity), AMPK (dilution of 1:1000, DF6361, Affinity), p-PI3K (dilution of 1:1000, AF3242, Affinity), PI3K (dilution of 1:1000, AF6241, Affinity), p-AKT (dilution of 1:1000, AF908, Affinity), AKT (dilution of 1:1000, AF6261, Affinity), and GAPDH (dilution of 1:5000; ab8245, Abcam) for overnight at 4 °C. Next, the membrane was washed four times with TBST for 10 min, incubated with Goat Anti-Mouse IgG (H+L) (dilution, 1:2000; SA00001-1; proteintech, China) or Goat Anti-Rabbit IgG (H+L) (dilution, 1:2000; SA00001-2; proteintech) secondary antibody for 1 h at RT, washed again four times, and visualized with Western ECL Substrate (Thermo Scientific) for chemiluminescence.

### Rat OA model

Male Sprague-Dawley (SD) rats (10 weeks old) were purchased from Army Medical University (Chongqing, China). All animal experiments were conducted in accordance with the guidelines of the Animal Experiments Committee and approved by the Institutional Review Board of Southwest Hospital. The experimental mice were subjected to surgically induced OA by anterior cruciate ligament transection (ACLT) as previously described [20]. The animals were divided into three groups: sham (*n* = 10), ACLT (*n* = 10), and ACLT+AA (*n* = 10). Rats in the ACLT+AA group were followed by intra-articular injection with 0.1 mL of AA (2.5 μg/mL) into the articular cavity once a week for 8 weeks, while rats in the ACLT group were injected with 0.1 mL of vehicle (0.9% NaCl) as a control. Food and water were available ad libitum. Rats were maintained under a constant temperature of 20 ± 2 °C, a relative humidity of 50% ± 10%, and a 12-h light/dark cycle. At 4 and 8 weeks post-AA intra-articular injection, 5 rats for each group were sacrificed, and knee joint tissues were collected for further evaluation.

### Macroscopic observation and histological analysis

Macroscopic evaluation was performed by five blinded investigators (LP, YJ, GL, FZ, and LR). The erosion of articular cartilage was graded according to the macroscopic score system [21].

Knee joint samples (*n* = 3) were fixed in 4% v/v paraformaldehyde for 2 days, and tissues were decalcified in 10% wt% EDTA disodium salt dihydrate (GRM1195, neofroxx, Germany) solution for 4 weeks at RT. After that, the samples were dehydrated through an alcohol gradient, cleared, and embedded in paraffin blocks. Frontal serial sections (4 μm thick) across entire joints were obtained and then stained with Safranin-O/Fast Green to assess cartilage destruction. The stained sections were photographed digitally under a microscope. Histologic changes in the medial tibial plateau and medial femoral condyle of knee joints were scored on a scale of 0–6 according to the recommendations of the Osteoarthritis Research Society International (OARSI) scoring system [22].

### Immunohistochemical analysis

Immunohistochemistry (IHC) was performed to evaluate the phenotype of articular chondrocytes. After deparaffinization and hydration with distilled water, the antigen repair was conducted at 37 °C for 10 min. Then, the tissue slices were penetrated with PBS for 5 min followed with H_2_O_2_ treatment for about 20 min, and then blocked for 60 min to avoid the homologous serum.

Sections were incubated overnight at 4 °C with antibodies against the following proteins: COL-II (dilution of 1:200), MMP-13 (dilution of 1:100), COL-X (dilution of 1:80), Runx2 (dilution of 1:500), COL-Ι (dilution of 1:200), and α-SMA (dilution of 1:200). After rinsing with PBS, sections were incubated with appropriate biotinylated secondary antibody and horseradish peroxidase-conjugated streptavidin-biotin. Immunoreactivity was visualized with a 3,3′-diaminobenzidine tetrahydrochloride kit (ZSGB Bio, China) followed by counterstaining with methyl green. The presence of antigen in the cartilage was estimated by calculating the number of chondrocytes that stained positive. The total number of chondrocytes and those that stained positive in three central regions of articular cartilage were counted using Image Pro Plus version 5.1 software (Media Cybernetics, USA). The percentage of positive stained cells for the antigen and the relative fold change of different groups were then determined.

### Statistical analysis

Data were expressed as mean ± standard deviation (SD). Statistical significance was assessed by one-way analysis of variance (ANOVA) (more than two groups) or unpaired *t* tests (two groups). *P* < 0.05 was considered statistically significant.

## Results

### Effects of AA on chondrocyte viability

**Fig. 1:**
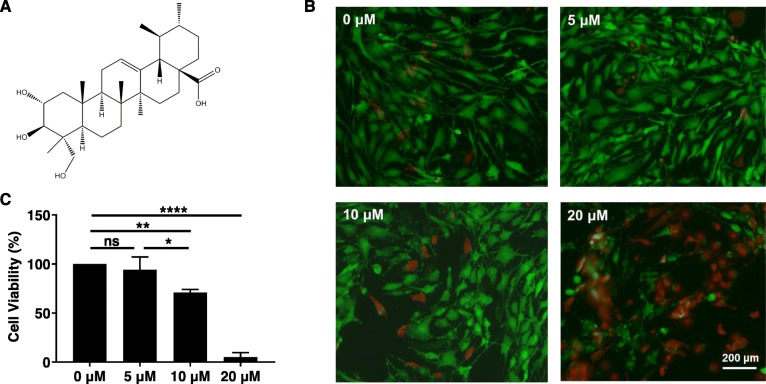
Effect of asiatic acid (AA) on chondrocyte viability. The cells were cultured with increasing concentrations of AA (0, 5, 10, and 20 μM) for 24 h. **a** The chemical structure of AA. **b** The live-dead cell staining of human osteoarthritic chondrocytes. **c** The cell viability was determined by CCK-8 assay. One-way ANOVA, *P < 0.05, **P < 0.01, ****P < 0.0001; ns, not significant. Each experiment was repeated three times

The chemical structure of AA is shown in Fig. 1a. The live-dead staining results of chondrocytes after AA treatment are shown in Fig. 1b. Chondrocytes treated with 5 μM AA showed comparable staining with untreated control, while 10 μM and 20 μM AA treatment induced more dead chondrocytes. CCK-8 assay (Fig. 1c) was used to quantitatively evaluate the viability of AA-treated chondrocytes. Consistent with live-dead staining results, 5 μM AA treatment did not affect chondrocyte viability (compared with control), while 10 μM (*P* < 0.01, compared with control) and 20 μM (*P* < 0.0001, compared with control) AA treatment significantly reduced chondrocyte viability. Therefore, 5 μM AA was used for the subsequent experiments.

### Effects of AA on hypertrophy marker expression in chondrocytes

**Fig. 2:**
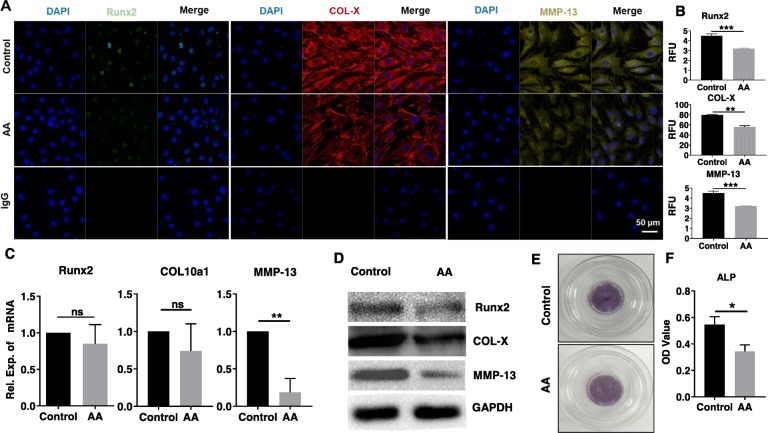
Effects of AA on hypertrophic marker expression of chondrocytes. The cells were treated with or without AA (5 μM) for 3 days. **a** Representative immunofluorescence images of Runx2 (green), COL-X (red), and MMP-13 (yellow). Nucleus was stained with DAPI (blue). **b** The RFU analysis of Runx2, COL-X, and MMP-13. Unpaired *t* test, **P < 0.01, ***P < 0.001. **c** Relative mRNA expression of Runx2, COL10a1, and MMP-13. Unpaired *t* test, **P < 0.01. ns, not significant. **d** Representative western blot of Runx2, COL-X, and MMP-13. GAPDH was served as a loading control. **e** Representative ALP staining of chondrocytes. **f** The OD value analysis of ALP. Unpaired *t* test, *P < 0.05. Each experiment was repeated three times

To analyze whether AA inhibited chondrocyte hypertrophy, we measured hypertrophic markers COL-X, MMP-13, and Runx2 using immunofluorescence staining, RT-PCR, and western blot analysis. In human OA chondrocytes, AA treatment reduced expression of Runx2, COL-X, and MMP-13 protein when compared to the control group, as demonstrated by immunofluorescence staining and its RFU analysis (Fig. 2a, b). RT-PCR results showed that the mRNA level of Runx2 and COL10a1 in the AA group was lower than that in the control group, but with no statistical difference (Fig. 2c); the mRNA level of MMP-13 in the AA group was significantly lower than that in the control group (*P* < 0.01). Western blot results further revealed that (Fig. 2d) the protein levels of Runx2, MMP-13, and COL-X in the AA group were reduced in different degrees, as compared with the control group. Additionally, a weaker staining of ALP after AA treatment was observed when compared to the control group (Fig. 2e, f). The reduction in ALP further confirmed the anti-hypertrophic effect of AA.

### Effects of AA on fibrosis factor expression in chondrocytes

**Fig. 3:**
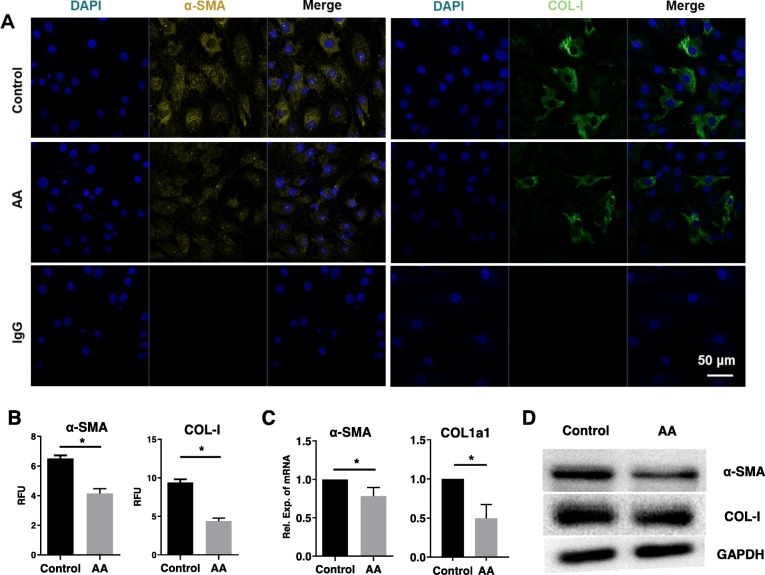
Effects of AA on fibrotic marker expression of chondrocytes. The cells were treated with or without AA (5 μM) for 3 days. **a** Representative immunofluorescence images of α-SMA (yellow) and COL-Ι (green). Nucleus was stained with DAPI (blue). **b** The RFU analysis of α-SMA and COL-Ι. Unpaired *t* test, *P < 0.05. **c** Relative mRNA expression of α-SMA and COL1a1. Unpaired *t* test, *P < 0.05. **d** Representative western blot of α-SMA and COL-Ι. GAPDH was served as a loading control. Each experiment was repeated three times

To establish if AA inhibited key regulators of chondrocyte fibrosis, the expression of COL-Ι and α-SMA was assessed. We found that the staining of COL-Ι and α-SMA in the AA group was weaker compared with the control group (Fig. 3a, b). COL1a1 mRNA level was significantly reduced by ~ 50% while α-SMA mRNA was significantly reduced by ~ 22% in the AA group compared with the control group (Fig. 3c). Consistently, protein (Fig. 3d) levels of COL-Ι and α-SMA were also significantly inhibited by AA treatment.

### Effects of AA on chondrocytic phenotype of chondrocytes

**Fig. 4:**
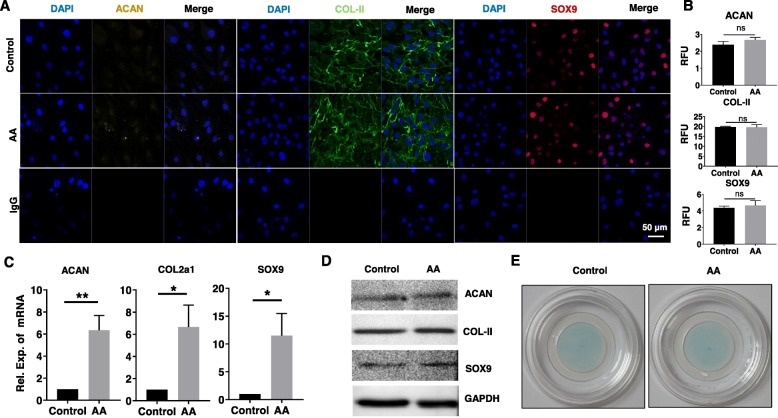
Effects of AA on chondrogenic marker expression of chondrocytes. The cells were treated with or without AA (5 μM) for 3 days. **a** Representative immunofluorescence images of ACAN (yellow), COL-II (green), and SOX9 (red). Nucleus was stained with DAPI (blue). **b** The RFU analysis of ACAN, COL-II, and SOX9. Unpaired *t* test; ns, not significant. **c** Relative mRNA expression of ACAN, COL2a1, and SOX9. Unpaired *t* test, *P < 0.05, **P < 0.01. **d** Representative western blot of ACAN, COL-II, and SOX9. GAPDH was served as a loading control. **e** Representative Alcian Blue staining of human OA chondrocytes. Each experiment was repeated three times

We further analyzed the effects of AA treatment on chondrocytic phenotype. It was shown that the immunofluorescence staining and its RFU analysis results of ACAN, COL-II, and SOX9 in the AA group were comparable with the control group (Fig. 4a, b). Although gene expression of COL2a1, ACAN, and SOX9 in the AA group was much higher than that in the control group (Fig. 4c), the protein levels of these markers were similar between the AA group and control group (Fig. 4d). In addition, the Alcian Blue staining showed no obvious difference between the control group and AA group as well (Fig. 4e).

### AA regulated the AMPK/PI3K/AKT signaling pathway

**Fig. 5:**
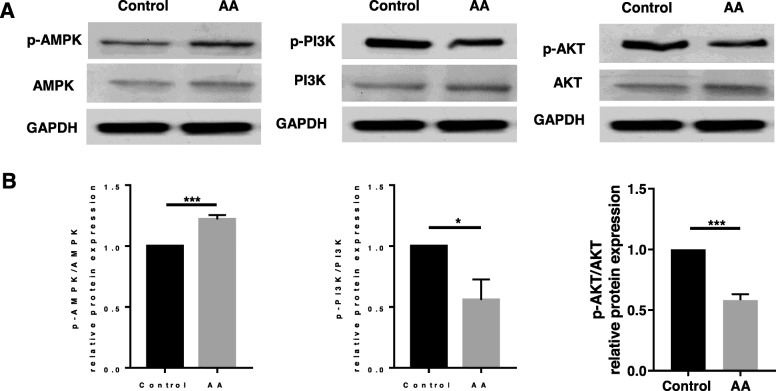
AA treatment activated the AMPK and inhibited PI3K/AKT signaling pathway. The cells were treated with or without AA (5 μM) for 3 days. **a** Representative western blot of p-AMPK, AMPK, p-PI3K, PI3K, p-AKT, and AKT. GAPDH was served as a loading control. **b** Relative protein expression of p-AMPK/AMPK, p-PI3K/PI3K, and p-AKT/AKT. Unpaired *t* test, *P < 0.05, ***P < 0.001. Each experiment was repeated three times (p-AMPK, phosphorylated AMP-activated protein kinase; AMPK, AMP-activated protein kinase; p-PI3K, phosphorylated phosphoinositide-3 kinase; PI3K, phosphoinositide-3 kinase; p-AKT, phosphorylated protein kinase B; AKT, protein kinase B)

To define further the molecular mechanism for the AA-mediated anti-hypertrophy and anti-fibrosis of human OA chondrocytes, we evaluated the potential role of the AMP-activated protein kinase (AMPK) and phosphoinositide-3 kinase/protein kinase B (PI3K/AKT) signaling pathway. After AA treatment, we found that the phosphorylation of AMPK was increased and the phosphorylation of PI3K and AKT was significantly decreased in the AA group (Fig. 5a) compared with the control group. Meanwhile, the ratio of phosphorylated AMP-activated protein kinase (p-AMPK)/AMPK was increased by ~ 23% and the ratios of phosphorylated phosphoinositide-3 kinase (p-PI3K)/PI3K and phosphorylated protein kinase B (p-AKT)/AKT were decreased by ~ 48% and ~ 40%, respectively (Fig. 5b). These results suggest that AA protected the chondrocytes against chondrocyte hypertrophy and chondrocyte fibrosis by activating AMPK and inhibiting the PI3K/AKT signaling pathway.

### Effects of AA on the cartilage destruction in ACLT rat model

**Fig. 6:**
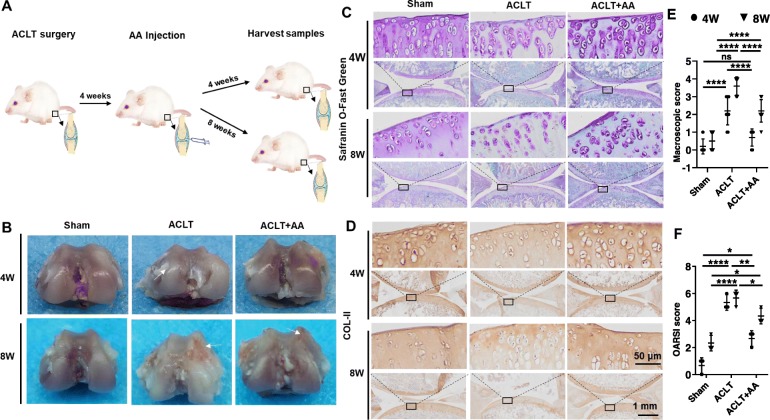
AA injection slows down cartilage degeneration in rat OA model. **a** Schematic of the ACLT-induced rat knee OA model. ACLT-induced rats treated with intra-articular (IA) injection of either vehicle or AA (2.5 μg/mL), and cartilage was isolated at 4 and 8 weeks. **b** Macroscopic appearance of cartilage from tibial plateaus of rats (*n* = 2 for each group). The arrow in the ACLT group at 4 weeks indicates cartilage hyperplasia, and the arrow in the ACLT group and ACLT+AA group at 8 weeks indicates eroded cartilage. **c** Safranin O/Fast Green stained sections of knee joints (*n* = 3 for each group). **d** Immunohistochemistry analysis of COL-II in sections of tibial plateau of knee joints (*n* = 3 for each group). **e** The macroscopic observation scores of knee joints (*n* = 2 for each group). **f** Cartilage degeneration evaluated with the Osteoarthritis Research Society International (OARSI) scoring system (*n* = 3 for each group). One-way ANOVA, *P < 0.05, **P < 0.01, ****P < 0.0001. Boxed regions in the bottom panels are shown in the top panels

The process of animal experiment is shown in Fig. 6a. Starting 1 month after the ACLT surgery, the following 4 weeks and 8 weeks were used as time points for observation. The knees in each group were isolated for macroscopic observation (Fig. 6b). Cartilage on the femoral condyles in the sham group appeared macroscopically normal with a smooth surface, and no cartilage defects or osteophytes were observed at 4 and 8 weeks. In the ACLT group, the cartilage surface was uneven and rough at 4 weeks and got eroded with local ulcers at 8 weeks. Interestingly, the cartilage surface in the ACLT+AA group was as smooth as that in the sham group.

Histological analysis of cartilage was performed using Safranin O-Fast Green staining (Fig. 6c). We found that the cartilage surface was smooth and showed positive red staining in the sham group. Compared with the ACLT group, the ACLT+AA group showed a milder severity of OA caused by ACLT surgery (Fig. 6c). The ACLT group showed cartilage erosion, apparent hypocellularity, and massive proteoglycan loss compared with the ACLT+AA group. Moreover, histochemical analysis showed that COL-II expression was decreased in the ACLT group compared with the sham and ACLT+AA group (Fig. 6d). The macroscopic observation scores and the OARSI scores were utilized to quantify the severity of cartilage damage (Fig. 6e, f). All these results suggested that AA slowed down the degeneration of cartilage matrix during OA development.

### Effects of AA on chondrocyte hypertrophy in rat OA model

**Fig. 7:**
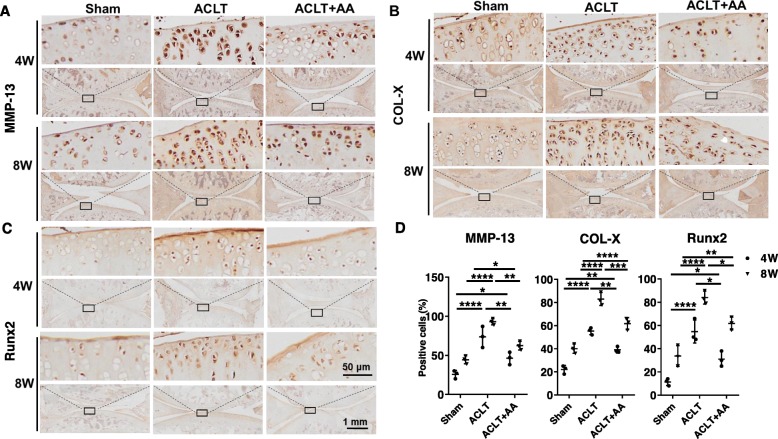
Effects of AA on chondrocyte hypertrophy in rats. **a**–**c** Immunohistochemistry analysis of and MMP13, COL-X, and Runx2 in sections of tibial plateau of knee joints. **d** Quantitative data of percentage of MMP13-, COL-X-, and Runx2-positive cells in each group (*n* = 3 for each group). One-way ANOVA, *P < 0.05, **P < 0.01, ***P < 0.001, ****P < 0.0001. Boxed regions in the bottom panels are shown in the top panels

Histochemical analysis revealed that ACLT operation induced COL-X, MMP-13, and Runx2 expression in the proximal tibia of articular cartilage of rats at 4 and 8 weeks post-intra-articular injection (Fig. 7a, c). Injection of AA reduced this induction, as was shown by the limited expression of COL-X, MMP-13, and Runx2 in the ACLT+AA group. The positive cells expressing COL-X, MMP-13, and Runx2 in the ACLT+AA group were much fewer than those in the ACLT group at 4 and 8 weeks (Fig. 7d). Taken together, these results indicated that AA attenuated the hypertrophy of chondrocytes in ACLT-induced rat OA model.

### Effects of AA on chondrocyte fibrosis in rat OA model

**Fig. 8:**
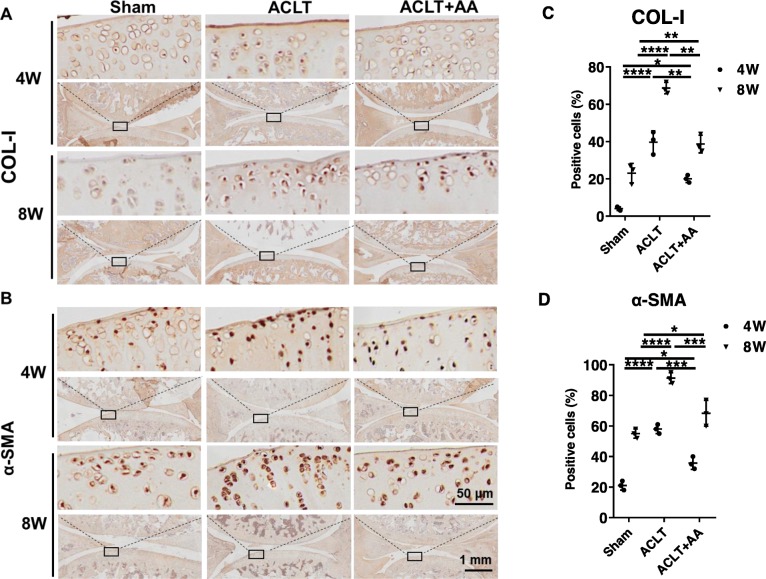
Effects of AA on chondrocyte fibrosis in rats. **a**, **b** Immunohistochemistry analysis of and COL-Ι and α-SMA in sections of tibial plateau of knee joints. **c** Quantitative data of percentage of COL-Ι- and α-SMA-positive cells in each group (*n* = 3 for each group). One-way ANOVA, *P < 0.05, **P < 0.01, ***P < 0.001, ****P < 0.0001. Boxed regions in the bottom panels are shown in the top panels

To evaluate the fibrosis of chondrocytes, histochemical analysis was performed to analyze the expression of COL-Ι and α-SMA in the proximal tibia of articular cartilage of rats at 4 and 8 weeks post-intra-articular injection (Fig. 8a, b). We found an elevated expression of COL-Ι and α-SMA expression in rat knee joints after the ACLT operation, but AA injection partly reversed the trend. In addition, quantitative data showed that the positive cells expressing COL-Ι and α-SMA in the ACLT+AA group were fewer than those in the ACLT group at 4 and 8 weeks (Fig. 8c, d). Taken together, these data revealed that AA repressed the chondrocyte fibrosis during OA development.

## Discussion

Articular chondrocytes tend to lose phenotypic stability when the microenvironment changes. In cartilage-related diseases such as OA, chondrocytes gradually acquire a hypertrophic phenotype; in in vitro culture, chondrocytes normally obtain a fibrotic phenotype. Therefore, how to avoid the unwanted phenotype and maintain the chondrogenic phenotype is a concern in chondrocyte biology. In this study, we found that AA could reduce chondrocyte hypertrophy and fibrosis via AMPK/PI3K/AKT signaling pathway.

First, we assayed the effect of AA on the human OA chondrocyte viability. Compared with 10 μM and 20 μM AA, 5 μM AA showed comparable staining with untreated control and less dead chondrocytes, which was further verified by CCK-8 assay. These results indicated that the range of concentration selection for future clinical use is relatively small, which needs more accurate understanding of mechanism to keep the precise control of dosage.

Then, we detected the change of hypertrophic markers COL-X, MMP-13, and Runx2 in human osteoarthritic chondrocytes after 5 μM AA treatment. As expected, AA downregulated the expression of COL-X, MMP-13, and Runx2 at protein level. Moreover, AA treatment induced a weaker ALP staining compared with untreated control. Similar with our results, Gu et al. found that resveratrol inhibited chondrocyte hypertrophy through suppressing the expression of MMP-13 in human articular chondrocytes [23]; Yahara et al. showed that Pterosin B prevented chondrocyte hypertrophy via downregulating the expression of COL-X, Runx2, and ALP [24].

In addition, we found that AA also effectively inhibited chondrocyte fibrosis. Note that in situ chondrocyte fibrosis tends to be connected with the onset of OA [25, 26]. The fibrotic remodeling of osteoarthritic cartilage enhanced by the presence of COL-I [27] and the secretion of α-SMA by osteoarthritic chondrocytes has been verified [28]. Our study showed that AA treatment lowered the expression of COL-I and α-SMA both in vitro and in vivo, indicating that the fibrotic differentiation of chondrocytes was attenuated by AA.

It has been shown that changes in collagen types from COL-II to COL-I, COL-III, and COL-X within OA cartilage participate to OA physiopathology by damaging the assembly, integrity, and stability of the extracellular matrix synthesized [29, 30]. Report on rabbit articular chondrocytes showed a final “dedifferentiated” phenotype, as 41% of COL-I, 25% of X_2_Y, 20% of COL-I trimer, 13% of COL-III, and 1% of COL-II [12]. In normal articular cartilage, COL-II is the more prominent collagen found associated in heteropolymer with COL-IX and COL-XI (∼ 1% COL-IX, ∼ 3% COL-XI, ≥ 90% COL-II). In accordance with these observations, authors proposed to consider COL-II/COL-I ratio as a signature for the dedifferentiation process [31]. These researches indicated that hypertrophic-like and fibroblastic-like phenotypes were increasing while chondrogenic phenotypes were decreasing during OA progression. Hence, we further studied the effects of AA treatment on the chondrogenic phenotype of chondrocytes. Chondrogenesis is characterized by the expression of SOX9, COL2a1, and ACAN. In conjunction with SOX5 and SOX6 [32] and interacting with CEBP/p300 [33], SOX9 activates the expression of chondrocyte-specific genes such as COL2a1 and ACAN, leading to deposition of COL-II and proteoglycans. However, we found that there was an increase expression of these chondrogenic markers (SOX9, COL-II, and ACAN) in the gene level but no significant difference in the protein level between AA-treated chondrocytes and untreated control. In fact, the mRNA abundance of a particular gene does not necessarily have a linear relationship with the expression of its translation product protein, because there are many levels of regulation of gene expression. mRNA degradation, protein degradation, modified folding, and other factors may lead to the expression difference between gene and protein level.

Based on these results, we further explored the mechanism of AA on anti-hypertrophic and anti-fibrosis in vitro. Previous studies have shown that certain signaling pathways are very important in regulating chondrocyte differentiation. For example, BMP2 and BMP4 induce hypertrophy during the chondrogenic differentiation of human MSCs in vitro [34]. In the presence of TGF-β, human MSCs show a significant enhancement of chondrogenesis and suppression of hypertrophic differentiation [35]. Moreover, postnatal WNT/β-catenin signaling in growth plate promotes hypertrophic differentiation and endochondral ossification [36]. Reinhold et al. indicated that canonical WNT signaling induces the transcription repressor Twist1, which strongly inhibits chondrocyte gene expression [37]. However, the role of PI3K/AKT pathway remains puzzling: some studies have reported a positive [38] while others a negative [39] effect on chondrocyte hypertrophy. In this study, we found that AA treatment attenuated chondrocyte hypertrophy and fibrosis through downregulating the phosphorylation of PI3K/AKT signaling pathway. In contrast, after activation of PI3K at the leading edge, AKT rapidly accumulates by binding to PtdIns (3,4,5) P_3_ via its pleckstrin homology domain, leading to activation of AKT by phosphorylation. The PI3K/AKT pathway was shown to be involved in Runx2-dependent osteoblast and enhanced DNA binding of Runx2 and Runx2-dependent transcription [38]. A previous study suggested that AKT enhances Runx2 protein stability by regulating Smurf2 function during osteoblast differentiation [40]. Another study also found that the PI3K/AKT pathway was required for hypertrophic differentiation. The major phenotype of the LY294002-treated tibiae was represented by a 45% reduction in the length of the hypertrophic zone [41]. In addition, AKT1 and AKT2, but not AKT3, were reported to inhibit fibrogenesis in hepatocytes and HSC [42]. Besides, previous studies have revealed that the PI3K/AKT signaling pathway is associated with hypertrophy and fibrosis in different tissues such as cardiac hypertrophy [43] and liver fibrosis [18]. Moreover, our data also found that AA treatment upregulated the phosphorylation of AMPK. A previous study has shown that the activation of AMPK could inhibit PI3K/AKT signaling via inhibition of PtdIns (3,4,5) P_3_ accumulation at the plasma membrane [44]. Hence, these results have indicated that AA might attenuate chondrocyte hypertrophy and fibrosis via AMPK-mediated PI3K/AKT signaling inhibition.

Interestingly, it is reported that loss of AMPK signaling changes downstream signaling and induces pro-inflammatory cytokines that potentially cause insulin resistance [45]. Another study shows that chondrocyte differentiation is functionally associated with decreased AMPK activity. Treatment with metformin, an activator of AMPK, significantly reduced cartilage matrix formation and inhibited gene expression of SOX6, SOX9, COL2a1, and aggrecan core protein (acp) [46]. However, our data showed no significant changes of chondrocytic phenotypes between the control group and AA-treated group, which indicated that AA might regulate the expression of chondrocytic phenotypes and keep SOX9 in balance via some other pathways. Future work is expected to explore the exact mechanism of AA on regulating chondrocyte differentiation. Beyond that, the study has shown that SOX9 can both interact directly with and suppress Runx2 activity and other genes related to hypertrophic chondrocytes, such as Col10a1 [47–49]. These researches implicated that AA might also alleviate hypertrophic differentiation via keeping the expression of SOX9 in balance.

**Fig. 9:**
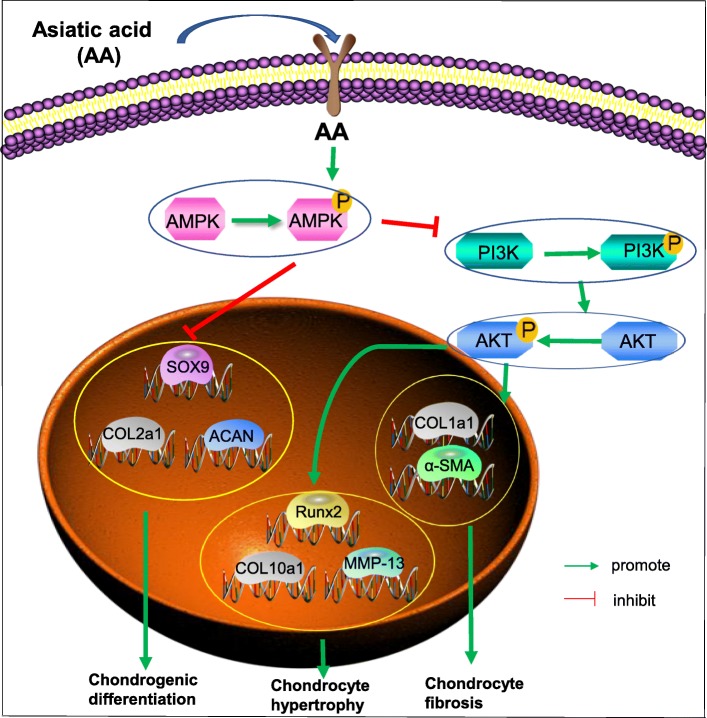
Schematic diagram of the mechanism of AA on attenuating hypertrophic and fibrotic differentiation

In our vivo experiment, the results showed that AA had a chondroprotective effect on ACLT rat model. Meanwhile, AA significantly reduced the expression of hypertrophic and fibroblastic phenotypes in both 4 and 8 weeks, which indicated that AA showed a curative effect on alleviating the OA progression. Taken together, these results reveal the modulation of AMPK/PI3K/AKT plays an essential part in the regulation of hypertrophy and fibrosis in human OA chondrocytes treated with AA in vitro (Fig. 9). It is worthy to note that ACLT surgery + AA injection was performed on relatively young rats in our study. In this case, the response of rat chondrocytes might not fully reflect the response of human osteoarthritic chondrocytes from aged patients to AA injection.

## Conclusions

In conclusion, AA treatment could reduce hypertrophic and fibrotic differentiation and maintain the chondrogenic phenotype of articular chondrocytes mainly via AMPK/PI3K/AKT signaling pathway. Our study suggested that AA might be a prospective drug component that targets hypertrophic and fibrotic chondrocytes for OA treatment.

## Data Availability

The datasets analyzed during the current study are available from the corresponding author on reasonable request.

## Abbreviations

OA: Osteoarthritis
AA: Asiatic acid
COL-X: Type X collagen
MMP-13: Matrix metalloproteinase-13
ALP: Alkaline phosphatase
Runx2: Runt-related transcription factor 2
COL-Ι: Type I collagen
α-SMA: Alpha-smooth muscle actin
SOX9: SRY-related HMG box 9
COL-II: Type II collagen
ACAN: Aggrecan
ACLT: Anterior cruciate ligament transection
p-AMPK: Phosphorylated AMP-activated protein kinase
AMPK: AMP-activated protein kinase
p-PI3K: Phosphorylated phosphoinositide-3 kinase
PI3K: Phosphoinositide-3 kinase
p-AKT: Phosphorylated protein kinase B
AKT: Protein kinase B
DMSO: Dimethylsulfoxide
OARSI: Osteoarthritis Research Society International
RT-PCR: Reverse transcription-polymerase chain reaction
SD: Standard deviation
ANOVA: Analysis of variance
RFU: Relative fluorescence unit
